# DNA Polymerase Gamma Recovers Mitochondrial Function and Inhibits Vascular Calcification by Interacted with p53

**DOI:** 10.7150/ijbs.65030

**Published:** 2022-01-01

**Authors:** Pengbo Wang, Boquan Wu, Shilong You, Saien Lu, Shengjun Xiong, Yuanming Zou, Pengyu Jia, Xiaofan Guo, Ying Zhang, Liu Cao, Yingxian Sun, Naijin Zhang

**Affiliations:** 1 Department of Cardiology, The First Hospital of China Medical University, 155 Nanjing North Street, Heping District, Shenyang, 110001, Liaoning Province, People Republic of China.; 2 College of Basic Medical Science, Institute of Translational Medicine, Key Laboratory of Medical Cell Biology, Ministry of Education, Key Laboratory of Liaoning Province, China Medical University, Shenyang, 110122, Liaoning Province, People Republic of China.

**Keywords:** DNA polymerase gamma, mitochondria, calcification, p53

## Abstract

DNA polymerase gamma (PolG) is the major polymerase of mitochondrial DNA (mtDNA) and essential for stabilizing mitochondrial function. Vascular calcification (VC) is common senescence related degenerative pathology phenomenon in the end-stage of multiple chronic diseases. Mitochondrial dysfunction was often observed in calcified vessels, but the function and mechanism of PolG in the calcification process was still unknown. The present study found PolG^D257A/D257A^ mice presented more severe calcification of aortas than wild type (WT) mice with vitamin D3 (Vit D3) treatment, and this phenomenon was also confirmed *in vitro*. Mechanistically, PolG could enhance the recruitment and interaction of p53 in calcification condition to recover mitochondrial function and eventually to resist calcification. Meanwhile, we found the mutant PolG (D257A) failed to achieve the same rescue effects, suggesting the 3'-5' exonuclease activity guarantee the enhanced interaction of p53 and PolG in response to calcification stimulation. Thus, we believed that it was PolG, not mutant PolG, could maintain mitochondrial function and attenuate calcification *in vitro* and* in vivo*. And PolG could be a novel potential therapeutic target against calcification, providing a novel insight to clinical treatment.

## Introduction

PolG is the major polymerase of mitochondrial DNA and responsible for replication and correction of mtDNA, to guarantee the expression of respiratory complexes related proteins synthesized by mtDNA and maintain mitochondrial function 1-3. Some previous studies indicated the quantity and quality of PolG could affect mitochondrial function, such as mitophagy, mitochondrial fusion and division, and mitochondria biogenesis 4-6. Especially various mutation of PolG affected mitochondrial quality control processes, leading to abnormal accumulation of defective mtDNA and mitochondrial dysfunction 2, 7-11. PolG D257A mutation mice was constructed as a mitochondria damaged model which the point mutation located in the proof-reading-deficient version of PolG and damaged 3'-5' exonuclease activity of polymerase, resulting in 3-fold to 5-fold increment of mutant mtDNA and increased amounts of deleted mtDNA 1. Some research also revealed PolG deficiency brought alteration to cellular metabolism 12, 13. Thus, PolG was recognized as a crucial stabilization factor of mitochondria, and any deficiency of PolG could damage mitochondrial function and eventually lead to cellular senescence induced by mitochondria.

VC is recognized as a frequent senescence related degenerative pathological phenomenon and is often observed in the end-stage of most chronic diseases, which is characterized by abnormal deposition of calcium and phosphorus in vascular walls 14-16. Meanwhile, calcification process was often accompanied with mitochondrial dysfunction and metabolism alteration. And the process could be affected by mitochondrial function via various cytokines depend manner such as p53 17. In addition, the development of calcification also could be regulated by mitochondria related behavior, such as mitophagy and apoptosis 18-22. And we also raised the hypothesis “Soil-Seed-Pest” model to summary how mitochondria were involved in the calcification process in previous study 23. PolG D257A mice was designed originally to imitate mitochondrial dysfunction, and also accompanied with a variety of senescence related degenerative phenotypes such as kyphosis and osteoporosis 1, 2. This special mutation also brought cardiac injury such as enlarged heart chamber and atherosclerosis, and mtDNA damage could further promote calcification of atherosclerotic plaques 24. However, the vascular calcification status of PolG D257A mice is unrevealed for now and little research has compared the effect of WT and mutant PolG on calcification. It's still uncertain whether PolG is involved in calcification process, and the specific molecular mechanisms of PolG remain to be ascertained.

In the present study, we performed *in vitro* and *in vivo* experiments to explore the potential mechanism of PolG in the calcification process. Our results suggested WT PolG could inhibit calcification process. We also found WT PolG could recruit and interact with p53 to stabilize mtDNA and mitochondrial function, and this interaction could be enhanced by beta-glycerophosphate (β-GP) induced calcification stimulation. However, PolG^D257A/D257A^ or mutant PolG seemly failed to activate p53-PolG complexes responding to calcification stimulation, eventually couldn't rescue mitochondrial function and attenuate calcification degree.

## Methods and Materials

### Reagents

Mouse anti-p53 (sc-126), mouse anti-BMP2 (sc-137087), mouse anti-PolG (sc-390634) and mouse anti-Runx2 (sc-101145) were purchased from Santa Cruz Biotechnology, Inc. (Dallas, TX, US). Rabbit anti-p53 (#10442-1-AP), rabbit anti-Runx2 (#20700-1-AP) and mouse anti-GAPDH (10494-1-AP) were purchased from Proteintech Group, Inc. (Rosemont, IL, USA). Rabbit anti-PolG (#13609) was purchased from Cell Signaling Technology, Inc. (Danvers, MA, USA). Horseradish peroxidase (HRP) goat anti-rabbit IgG (AS014), HRP goat anti-mouse IgG (AS003) were purchased from ABclonal Biotech, Co., Ltd. (Wuhan, China). Rabbit anti-alpha Tublin (WL02296) and rabbit anti-COX4 (WL02203) were purchased from Wanleibio, Ltd. (Shenyang, China).

### Cell line culture and *in vitro* calcification model

Vascular smooth cells (VSMCs) were cultured at 37°C in Dulbecco's modified Eagle's medium (DMEM, biological Industries, Cromwell, CT) supplemented with 10% fetal bovine serum (FBS, Clark Bioscience, Richmond, VA), 100 U/ml penicillin and 100 μg/ml streptomycin (Genview, Florida, USA), in a 37°C incubator with a humidified, under 5% CO2 atmosphere.

To induce the calcification phenotype of VSMCs *in vitro*, we treated VSMCs with calcification medium contained 1% FBS and 10mM β-GP when VSMCs were 80% reaching confluence for 5 days, and change the calcification medium every 2 days.

### Animal and *in vivo* calcification model

The PolG^D257A/D257A^ mice were a generous gift from Professor Nils-Göran Larsson 1. Mice were maintained in a specific pathogen-free and 21-23°C facility, fed with a standard chow diet (LabDiet, 5C02C). The genotyping was performed by standard polymerase chain reaction (PCR) with primer as forward: 5'-GCCTCGCTTTCTCCGTGACT-3' and reverse: 5'- GGATGTGGCCCAGGCTGTAACTCA-3'. All animal experiments were approved and performed in accordance with the guidelines of the Institutional Animal Care and Use Committee (IACUC) of China Medical University.

To induce the *in vivo* calcification phenotype of vascular, we used the classic and recognized protocol of mouse calcification model 25. The mice were injected subcutaneously Vit D3 solution [8*10^5 UI/Kg Vit D3 (#V8070, Solarbio, China) mixed in corn oil (#C7030, Solarbio, China)] last for 3 days, and measured the weight every 3 days until sacrifice in day 12.

### Plasmid construction, transfection, viral infection and gene knockout by CRISPER/Cas-9

To imitate PolG D257A mice, we constructed mutant PolG plasmid. The PolG mutation plasmid was constructed by Quick Change Site- Directed Mutagenesis Kit (Stratagene, La Jolla, CA) and the mutagenesis primers sequence were 5'-CACAATGTTTCCTTTGCCCGAGCTCATATCAGG-3' for forward primer and 5'-CCTGATATGAGCTCGGGCAAAGGAAACATTGTG-3' for reverse primer.

For plasmid transfection, the cells were plated around 60% reaching confluence before transfection, and next day assembled and incubated the transfection mixture which contained plasmid and Lipofectamine 2000 (#11668019, Thermo Fisher Scientific) and phosphate buffered saline (PBS) balance solution (Biological Industries, Cromwell, CT) in 37 ℃ for 15 minutes and then added the mixture into the cells medium, changed the medium 6h later. The cells were harvested in 48h after transfection. Additional, the detail method about transfection PolG and p53 to p53 knockout and PolG knockdown VSMCs in β-GP induced calcification model respectively were following. First, assemble the transfection system conclude plasmid of PolG or p53, PBS solution and Lipofectamine 2000. To guarantee the sufficient expression of PolG or p53 in mitochondria, the quality of plasmids is over saturated (10ug per 10cm dish). After incubating the system in 37 ℃ for 15 minutes, adding the system into the medium of cells and changing the medium after 6 hours. Then, 24 hours after transfection, replace the medium with calcified medium for culture and change the calcified medium every two days. On the 3rd day of the whole process, use system and method mentioned before to transfect the cells again. The cells are harvested on the 5th day for the next experimental study.

For viral infection, the lentiviral shRNA against human PolG was purchased from GeneChem (Shanghai, China). The shRNA sequence was 5′-TGTCCAGGGAGAGTTTATA-3′. A scrambled shRNA was included as a negative control (NC). Stable PolG silencing cell line and NC cell line were selected out with 10 μg/mL puromycin for 5 days after infection.

For p53 knockout VSMCs, we performed CRISPER/Cas-9 technology to achieve knockout. In brief, we chose sgRNA as following, 5'-CACCGTCGACGCTAGGATCTGACTG-3' and 5'- AAACCAGTCAGATCCTAGCGTCGAC-3'. After transfecting into VSMCs for 2 days and treated with puromycin to filtrate positive cells, then cultured monoclonal cell and sequenced the DNA of them, eventually harvested p53 knockout cell lines.

### Western blot (WB) analysis

Detailed protocol was description in previous studies 26, 27. In brief, cells or vascular tissues were lysed on the ice for 30 min with lysis buffer [50 mM Tris/HCl (pH 7.4), 150 mM NaCl, 1% NP‑40, 1% Triton X‐100, 0.25% sodium deoxycholate, 1 mM EDTA and protease inhibitor cocktail (B14001, Bimake, Houston, TX, USA)]. Vortex the lysates every 10 min and then centrifuged them at 13000g for 20min at 4°C. Then pipetted the supernatant contents contained protein and used G250 to measure the concentration, and prepared 30-50μg lysates for blotting. Load the samples into 8% or 12% polyacrylamide gels, and then separated by SDS-PAGE and transferred to PVDF membranes (ISEQ00010, Millipore) under 80v for 150min. Then blocked the membranes with 5% nonfat milk in TBST solution [20 mM Tris (pH 7.4), 137 mM NaCl and 0.05% Tween‑20] for 1h at room temperature, and probed with primary antibodies overnight at 4°C. The next day, after washing membranes with TBST solution 3 times, the membranes were incubated with second antibodies for 1h at room temperature. Finally, the membranes were detected by chemiluminescence (Tanon, Tanon Science & Technology Co., Ltd., Shanghai, China).

### Co-immunoprecipitation (CO-IP) assay

As usual protocol we conducted before^28^, VSMCs or tissues were harvested and then lysed on the ice for 30min, then centrifuged the lysates at 13000g for 20min at 4°C and pipetted the supernatant. Next, coupled the primary antibody with protein A/G beads (sc-2003, Santa Cruz Biotechnology, US), and added the immune complex into the lysates on a rocking platform overnight at 4°C.

After immunoprecipitation, the beads were washed with lysis buffer for 3 times and eluted the protein contents with 2× SDS sample buffer. Then took the sample to conduct WB analysis which was mentioned before.

### Alizarin Red staining

The cells were washed 3 times with PBS solution and then fixed with 4% paraformaldehyde (#AR-0211, Dingguo Changsheng Biotechnology CO. Ltd, China) for 30min at room temperature, then washed the cells with PBS solution and stained with 2% Alizarin red, pH 4.2 (#G1452, Solarbio, China) for 30min in 37°C incubator. Finally, rinsed the stained cells with distilled water for 3 times at room temperature and observed under the microscope.

### Von-kossa staining

Mice were sacrificed at 12 days after first injection. Isolated the aortas and fixed in 4% paraformaldehyde overnight at 4°C, and then put the fixed aortas into 5%, 10%, 20% and 30% sucrose solution. Embedded the aortas with OCT (#4583, Sakura, US) and cut into 10 μm section. The following staining protocol was performed by Von-kossa staining kit (#G3282, Solarbio, China). In brief, washed the sections with distilled water for 30 min, then incubated with silver nitrate solution and exposed with UV for 30min, finally treated the sections with sodium thiosulfate solution for 10 minutes at room temperature and observed under the microscope.

### Quantification of calcium deposition

Washed the cells or the vascular tissues with PBS solution and then decalcified with 0.6M HCL in 37°C incubator for 12 hours, pipetted the supernatant and used quantification detection of calcium kit (#BC0720, Solarbio, China) to measure the calcium ions level. Meanwhile, added lysis buffer (0.1M NaOH, 0.1% SDS) to the cells or tissues and lysed for 30min, then centrifuged at 13000g for 20min at 4°C, used BCA kit (#P0012, Beyotime, China) to measure the protein concentration to standardized the calcium level.

### Determination of mtDNA copy number

Total RNA was extracted from cells or tissues by Trizol method according to instructions (Ambion, US). The reverse transcription was conducted by PrimeScript™ RT Master Mix kit (RR036A, Takara. Co, Shiga, Japan) and the real time PCR was performed by TB Green® Premix Ex TaqTM II kit (RR820A, Takara. Co, Shiga, Japan) and the protocol was 30sec at 95°C followed by 40 cycles of 5sec at 95°C and 30sec at 60°C. We detected ND-1 levels present mtDNA copy numbers and used GAPDH as reference. The primers used for real time PCR was following, forward primer for ND-1: 5'-CCCTAAAACCCGCCACATCT-3' and reverse primer for ND-1: 5'-GAGCGATGGTGAGAGCTAAGGT-3', forward primer for GAPDH: 5-GCACCGTCAAGGCTGAGAAC-3 and reverse primer for GAPDH: 5-TGGTGAAGACGCCAGTGGA-3.

### ATP contents determination

ATP concentration was measured by ATP contents measurement kit according to the manufacturer's instructions (#A095-1-1, Nanjing Jiancheng Biological Engineering Institute, China). In brief, added distilled water to the cells or fresh tissues at boiled water atmosphere for 2min, and pipetted supernatant into mix solution which produced in the kit and incubated for 30min at 30°C, then mixed the mixture and terminate solution and pipetted out 200μl to measure at 592 nm by scanning with an absorbance reader (TECAN, Switzerland). The concentration of ATP for each sample was calculated using an ATP standard curve. In addition, protein concentration was determined using the G250 to standardized the ATP concentrations.

### Mitochondrial membrane potential (MMP) determination

MMP measurement kit with JC-1 was used according to the manufacturer's instructions (#M8650, Solarbio, China). The process conducted in a dark room, after incubating with fluorescent JC-1 at 37°C, the cells were washed 3 times with PBS and suspended in JC-1 balance buffer. In normal condition, JC-aggregates formed red fluorescence, while injured mitochondria present decline potential and the JC-1 monomer formed green fluorescence, the alteration of MMP was calculated as the red/green fluorescent intensity ratio.

### MBP (mean blood pressure) measurement

The mice were placed at a suitable temperature, and the tail was placed in the blood pressure measuring element (BP-2010, Softron, China). Measured the MBP of tail artery when the mice were in a. Each mouse repeated the measurement three times, with an interval of 2 minutes between each measurement. The mean of the three times was taken as the MBP of the mouse for following study.

### Statistical analysis

Data are presented as the mean ± standard deviation (SD) and were assessed for homogeneity of variance by the F-test and Brown-Forsythe test for group pairs and multiple groups, respectively. Data normality was examined by the Shapiro-Wilk test. Student's and Welch's t-tests were applied for data with equal and unequal variances, respectively (group pairs). One- and two-way ANOVAs were performed to assess differences in multiple groups, which involved one and two factors, respectively, with a Bonferroni post-hoc test. P-value adjustment was performed for multiple comparisons as appropriate. Data analysis was carried out using SPSS 22.0 (SPSS, USA) with P < 0.05 deemed statistically significant.

## Results

### PolG is involved in β-GP induced calcification

**Figure 1:**
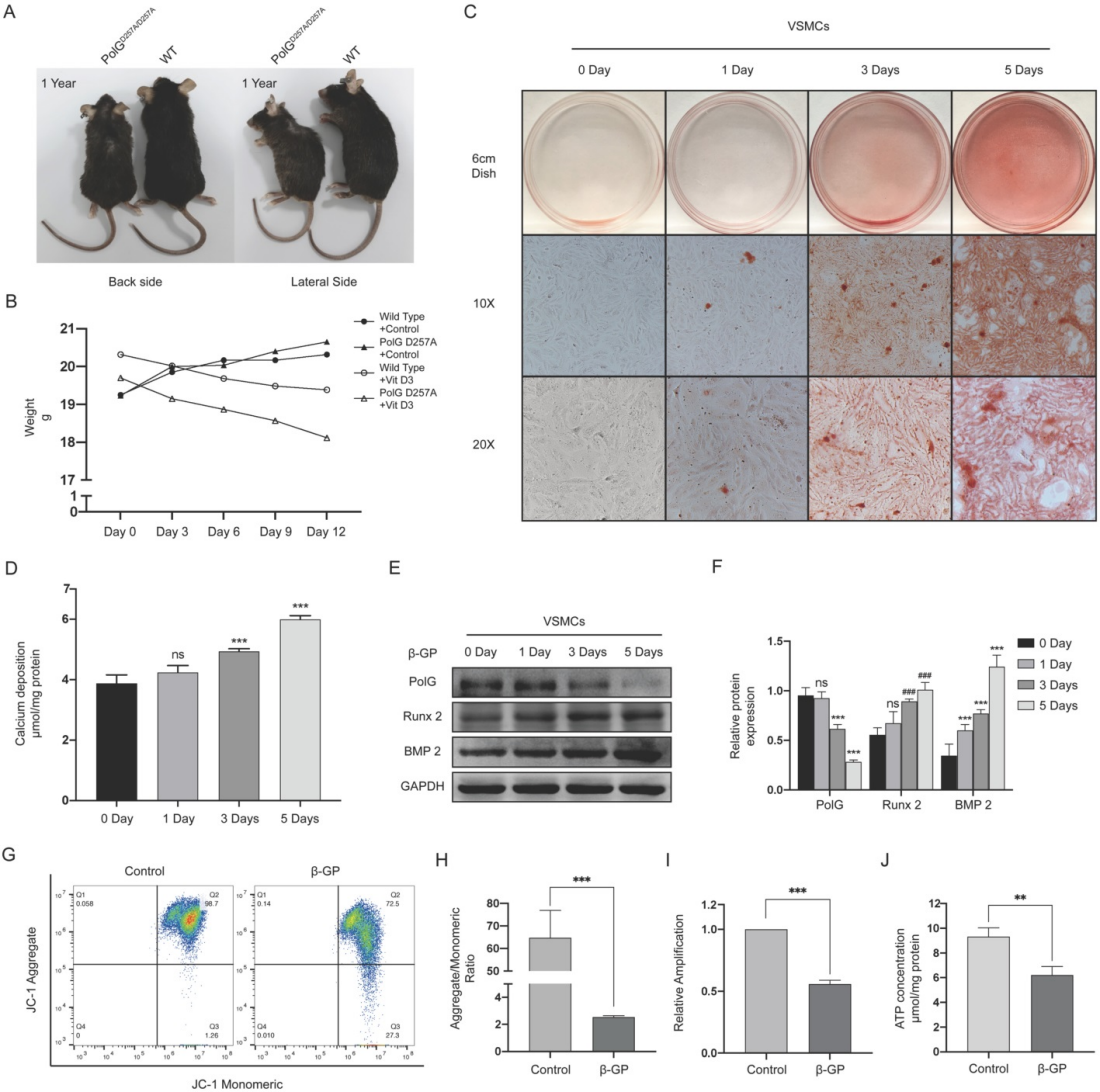
** PolG is involved in β-GP induced calcification.** (A) Different of wild type mice and PolG^D257A/D257A^ mice. (B) Weight curve of wild type mice and PolG^D257A/D257A^ mice with or without Vit D3 treatment, the weight was recorded every 3 days. (C) Alizarin red staining of VSMCs with 10mmol β-GP treated in different days. (D) Quantification of calcium deposition of VSMCs with β-GP treated in different days, expressed as the mean ± SD. (E, F) Western blot analysis and quantification of protein expression in VSMCs treated with 10mmol β-GP (PolG, Runx 2, BMP 2 and GAPDH), GAPDH was used as a loading control, and the day 0 was set as control in each parameter. ***p < .001, **p < .01 *p < .05, ^###^p < .001, ^##^p < .01, ^#^p < .05, ns p>0.05. Data were expressed as the mean ± SD of triplicate experiments. (G, H) VSMCs was treated with or without 10mmol β-GP for 5 days, used flow cytometry to detect MMP level of mitochondria. The potential alteration was calculated as red/green fluorescent intensity ratio. Data were collected from 3 repeat experiment. ***p < .001. (I) mtDNA copy numbers of VSMCs treated with or without 10mmol β-GP for 5 days. Data were collected from 3 repeat experiment and expressed as the mean ± SD. ***p < .001. (J) ATP contents of VSMCs treated with or without 10mmol β-GP for 5 days. Data were collected from 3 repeat experiment and expressed as the mean ± SD. **p < .005.

Compared with WT mice, the PolG^D257A/D257A^ mice presented significant senescence related degenerative alteration such as reduced body size, kyphosis and alopecia (Figure 1A). And we found PolG^D257A/D257A^ mice presented significantly decline in body weight than WT mice under treatment with Vit D3(Figure 1B). In addition, we treated VSMCs with 10 mM β-GP for 0, 1, 3 and 5 days to imitate *in vitro* calcification, and conducted alizarin red staining and quantification of calcium deposition to measure the calcification level. We found β-GP could induced VSMCs calcification in a time-dependent manner (Figure 1C, D). We also detected the calcification related protein expression and draw similar tendency with before which runt-related transcription factor 2 (Runx 2) and bone morphogenetic protein 2 (BMP 2) were gradually increased, but we also observed the expression of PolG was gradually decreased with the stimulation time (Figure 1E. F). Besides, we performed MMP detection, quantification of mtDNA copy numbers and ATP contents determination assay to evaluate mitochondrial function. Under the condition of calcification stimulation, we observed decreased MMP, decline in mtDNA copy numbers and ATP concentration which indicated mitochondrial function was damaged by calcification (Figure 1G-J).

These results suggested PolG was involved in the process of calcification via stabilizing mitochondrial function.

### PolG could stabilize mitochondrial function and alleviate calcification

**Figure 2:**
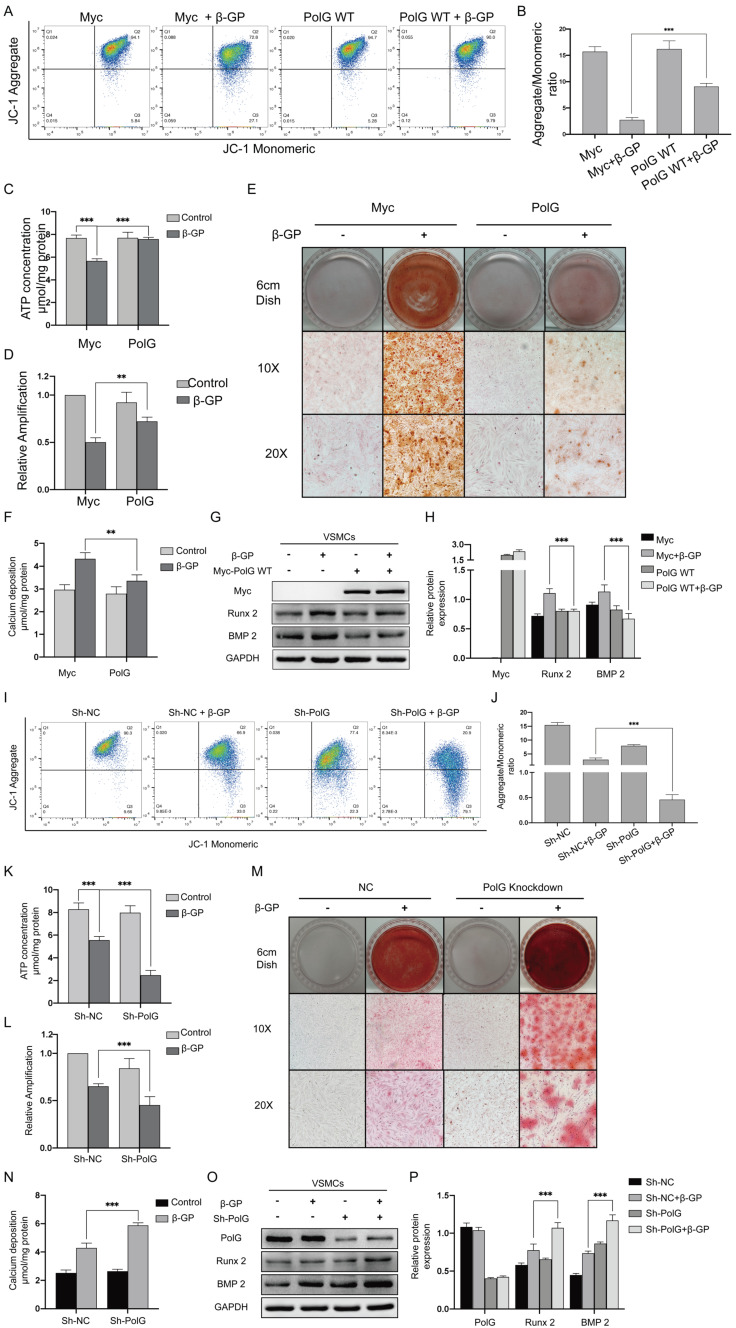
** PolG could stabilize mitochondrial function and alleviate calcification.** (A-H) VSMCs was transfected with Myc-WT PolG in 10mmol β-GP induced calcification for 5 days, and then conducted related experiment to detect mitochondrial function and calcification status. (A, B) Used flow cytometry to detect MMP level of mitochondria. The potential alteration was calculated as red/green fluorescent intensity ratio. Data were collected from 3 repeat experiment. ***p < .001. (C) mtDNA copy numbers of VSMCs. Data were collected from 3 repeat experiment and expressed as the mean ± SD. ***p < .001. (D) ATP contents of VSMCs. Data were collected from 3 repeat experiment and expressed as the mean ± SD. **p < .005. (E) Alizarin red staining of VSMCs. (F) Quantification of calcium deposition of VSMCs, data were collected from 3 repeat experiment and expressed as the mean ± SD. ***p < .001. (G, H) Western blot analysis and quantification of protein expression (Myc-tag, Runx 2, BMP 2 and GAPDH), GAPDH was used as a loading control, and the VSMCs transfected with Myc-tag vector and without treated with β-GP was set as control in each parameter. ***p < .001. Data were expressed as the mean ± SD of triplicate experiments. (I-P) Treated PolG knockdown or negative control VSMCs with or without 10mmol β-GP for 5 days, and then conducted related experiment to detect mitochondrial function and calcification status. (I, J) Used flow cytometry to detect MMP level of mitochondria. The potential alteration was calculated as red/green fluorescent intensity ratio. Data were collected from 3 repeat experiment. ***p < .001. (K) mtDNA copy numbers of VSMCs. Data were collected from 3 repeat experiment and expressed as the mean ± SD. ***p < .001. (L) ATP contents of VSMCs. Data were collected from 3 repeat experiment and expressed as the mean ± SD. **p < .005. (M) Alizarin red staining of VSMCs. (N) Quantification of calcium deposition of VSMCs, data were collected from 3 repeat experiment and expressed as the mean ± SD. ***p < .001. (O, P) Western blot analysis and quantification of protein expression (PolG, Runx 2, BMP 2 and GAPDH), GAPDH was used as a loading control, the PolG knockdown VSMCs and without treated with β-GP was set as control in each parameter. ***p < .001. Data were expressed as the mean ± SD of triplicate experiments.

To further reveal the role of PolG in the calcification process, we overexpressed and knockdown PolG in VSMCs calcification model. We observed overexpression of PolG could significant partly recovered decreased MMP, mtDNA copy numbers and production of ATP (Figure 2A-D). Meanwhile, the alizarin red staining and calcium deposition status shown overexpression of PolG significant inhibited calcification induced by β-GP (Figure 2E-F). In addition, we also find PolG could decrease the expression of calcification markers Runx 2 and BMP 2 (Figure 2G-H). While, knockdown of PolG had opposite effects, aggravating the mitochondrial dysfunction and calcification induced by β-GP (Figure 2I-P).

These alterations suggested PolG might play a crucial role in stabilization mitochondrial function and relieve calcification status.

### Mutant PolG is fail to stabilize mitochondrial function and against calcification

**Figure 3:**
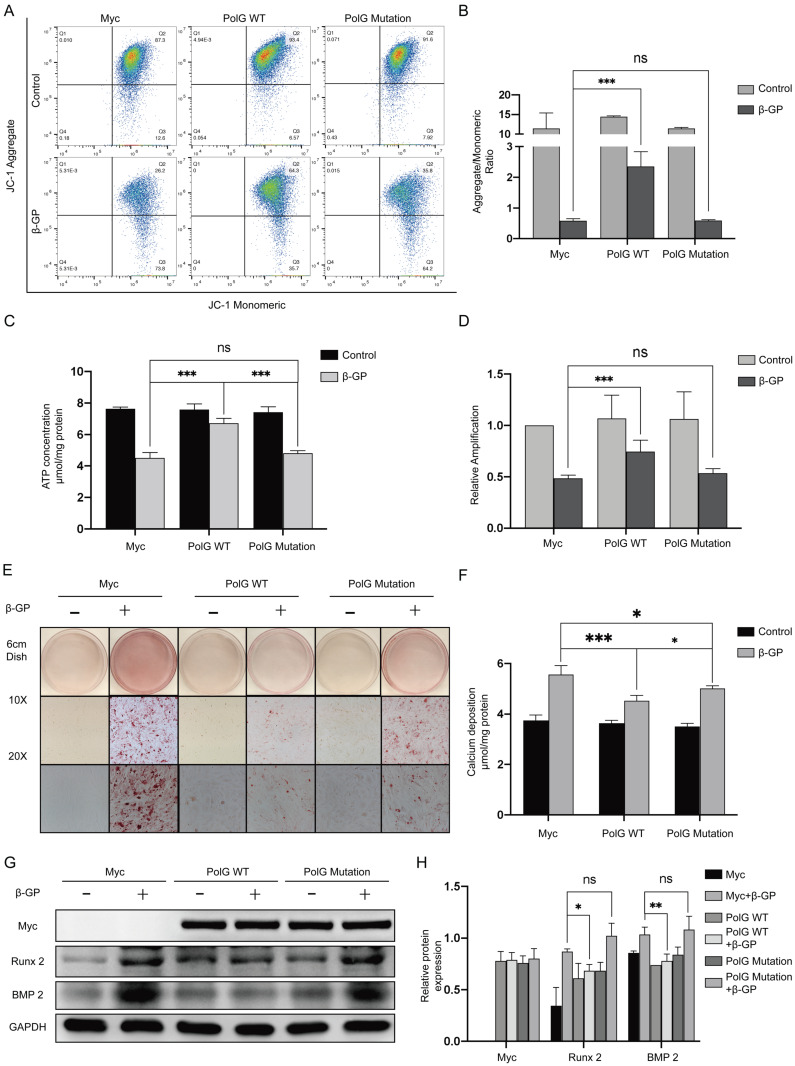
** Mutant PolG is fail to stabilize mitochondrial function and against calcification.** VSMCs was transfected with Myc-vector, WT PolG and mutant PolG with or without 10mmol β-GP induced calcification for 5 days, and then conducted related experiment to detect mitochondrial function and calcification status. (A, B) Used flow cytometry to detect MMP level of mitochondria. The potential alteration was calculated as red/green fluorescent intensity ratio. Data were collected from 3 repeat experiment. ***p < .001, ns p>0.05. (C) mtDNA copy numbers of VSMCs. Data were collected from 3 repeat experiment and expressed as the mean ± SD. ***p < .001, ns p>0.05. (D) ATP contents of VSMCs. Data were collected from 3 repeat experiment and expressed as the mean ± SD. ***p < .001, ns p>0.05. (E) Alizarin red staining of VSMCs. (F) Quantification of calcium deposition of VSMCs, data were collected from 3 repeat experiment and expressed as the mean ± SD. ***p < .001, ns p>0.05. (G, H) Western blot analysis and quantification of protein expression (Myc-tag, Runx 2, BMP 2 and GAPDH), GAPDH was used as a loading control, and the VSMCs transfected with Myc-tag vector and without treated with β-GP was set as control in each parameter. ***p < .001. Data were expressed as the mean ± SD of triplicate experiments.

In order to further clarify the mechanism of PolG in process of calcification, after transfecting WT and mutant PolG plasmids to VSMCs treated with β-GP, we found mutant PolG couldn't restore decreased MMP or recover declined mtDNA copy numbers and ATP production, it seemed mutant PolG couldn't protect mitochondria from calcification stimulation unlike what WT PolG did (Figure 3A-D). And corresponding, we also observed mutant PolG was fail to relieve β-GP induced calcification. Alizarin red staining shown WT PolG could greatly alleviate calcification degree, while mutant PolG just partly relieved calcification condition (Figure 3E). As for calcium deposition quantification determination and the expression of Runx 2 and BMP 2, we didn't find effects of mutant PolG on relieving calcification (Figure 3F-H).

The above results indicated mutant PolG was fail to stabilize mitochondrial function and relieve calcification.

### PolG deficiency induced aggravated calcification can be rescued by WT PolG but not the mutant PolG

**Figure 4:**
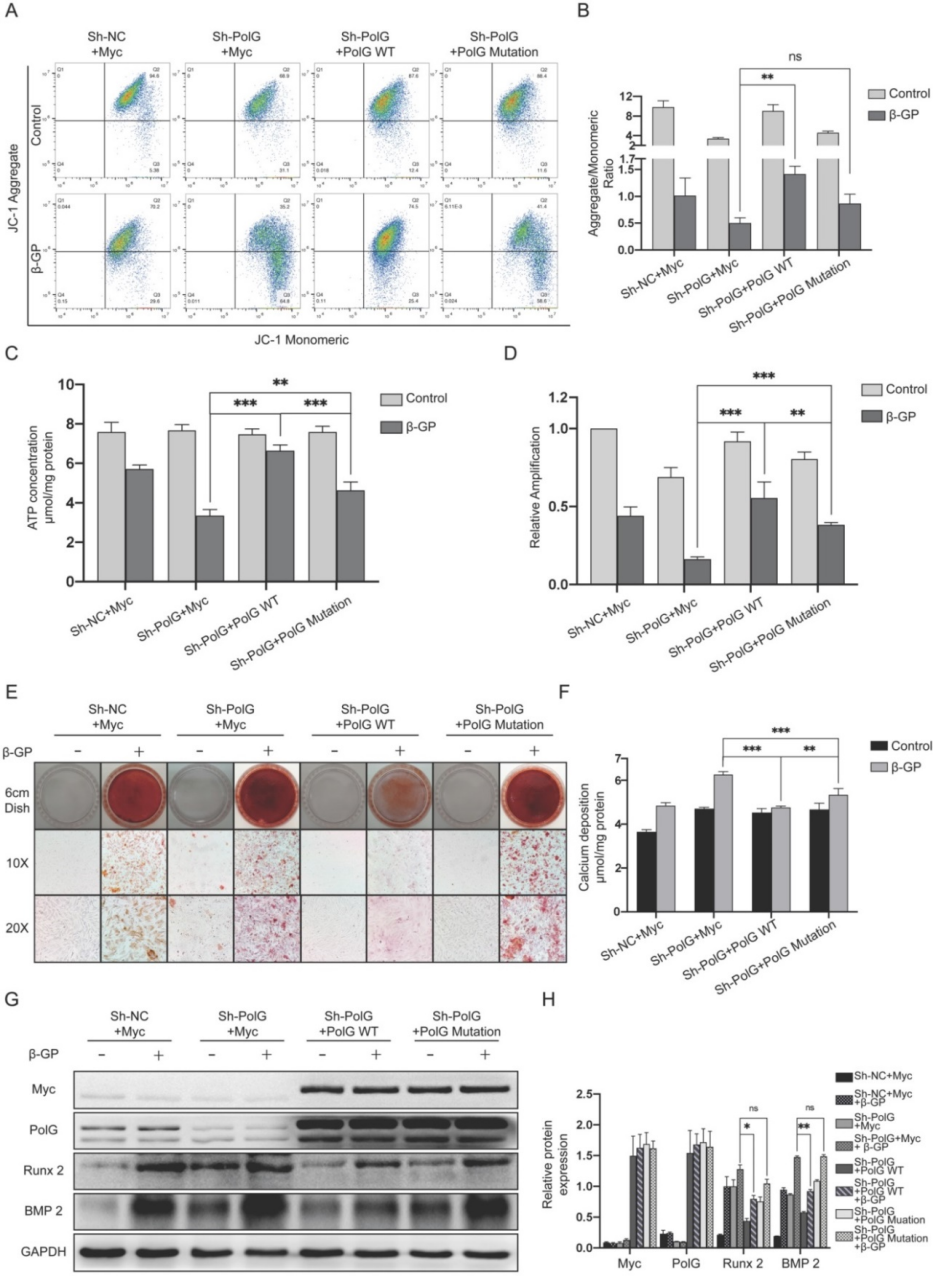
** PolG deficiency induced aggravated calcification can be rescued by WT PolG but not the mutant PolG.** NC VSMCs was transfected with Myc-vector and PolG knockdown VSMCs were transfected with Myc-vector, WT PolG or mutant PolG. All groups were treated with or without 10mmol β-GP for 5 days, and then conducted related experiment to detect mitochondrial function and calcification status. (A, B) Used flow cytometry to detect MMP level of mitochondria. The potential alteration was calculated as red/green fluorescent intensity ratio. Data were collected from 3 repeat experiment. **p < .005, ns p>0.05. (C) mtDNA copy numbers of VSMCs. Data were collected from 3 repeat experiment and expressed as the mean ± SD. ***p < .001, **p < .005. (D) ATP contents of VSMCs. Data were collected from 3 repeat experiment and expressed as the mean ± SD. ***p < .001, **p < .005. (E) Alizarin red staining of VSMCs. (F) Quantification of calcium deposition of VSMCs, data were collected from 3 repeat experiment and expressed as the mean ± SD. ***p < .001, **p < .005. (G, H) Western blot analysis and quantification of protein expression (Myc-tag, PolG, Runx 2, BMP 2 and GAPDH), GAPDH was used as a loading control, and the NCVSMCs transfected with Myc-tag vector and without treated with β-GP was set as control in each parameter. **p < .005. *p < .05, ns p>0.05. Data were expressed as the mean ± SD of triplicate experiments.

To further reveal the different effects on rescuing mitochondrial function and relieving calcification damage between WT and mutant PolG, we transfected them to PolG knockdown cell line respectively. We found PolG deficiency brought more severe mitochondria damage, but WT PolG could greatly rescue the mitochondrial function to normal level or even better, no matter the MMP or mtDNA copy numbers and ATP production. While, we still observed mutant PolG could only recover the mitochondrial function a little better than PolG knockdown cell line and couldn't reach the mitochondrial level of WT VSMCs (Figure 4A-D). This difference was more significant in calcification degree of VSMCs. WT PolG could greatly attenuate the degree of calcification and rescue the aggravation of calcification caused by PolG deficiency whether it is the level of calcium deposition or the calcification molecule level of calcification (Figure 4E-H). While we only observed mutant PolG could reduce calcium deposition, but we didn't find a rescue effect on calcification in alizarin red staining and expression of Runx 2 and BMP 2 detection compared with PolG knockdown VSMCs (Figure 4E-H).

These above results suggested PolG deficiency induced aggravated calcification could be greatly rescued by WT PolG, but mutant PolG could hardly rescue damage induced by PolG deficiency.

### The stabilization of mitochondrial function by PolG is regulated by p53

**Figure 5:**
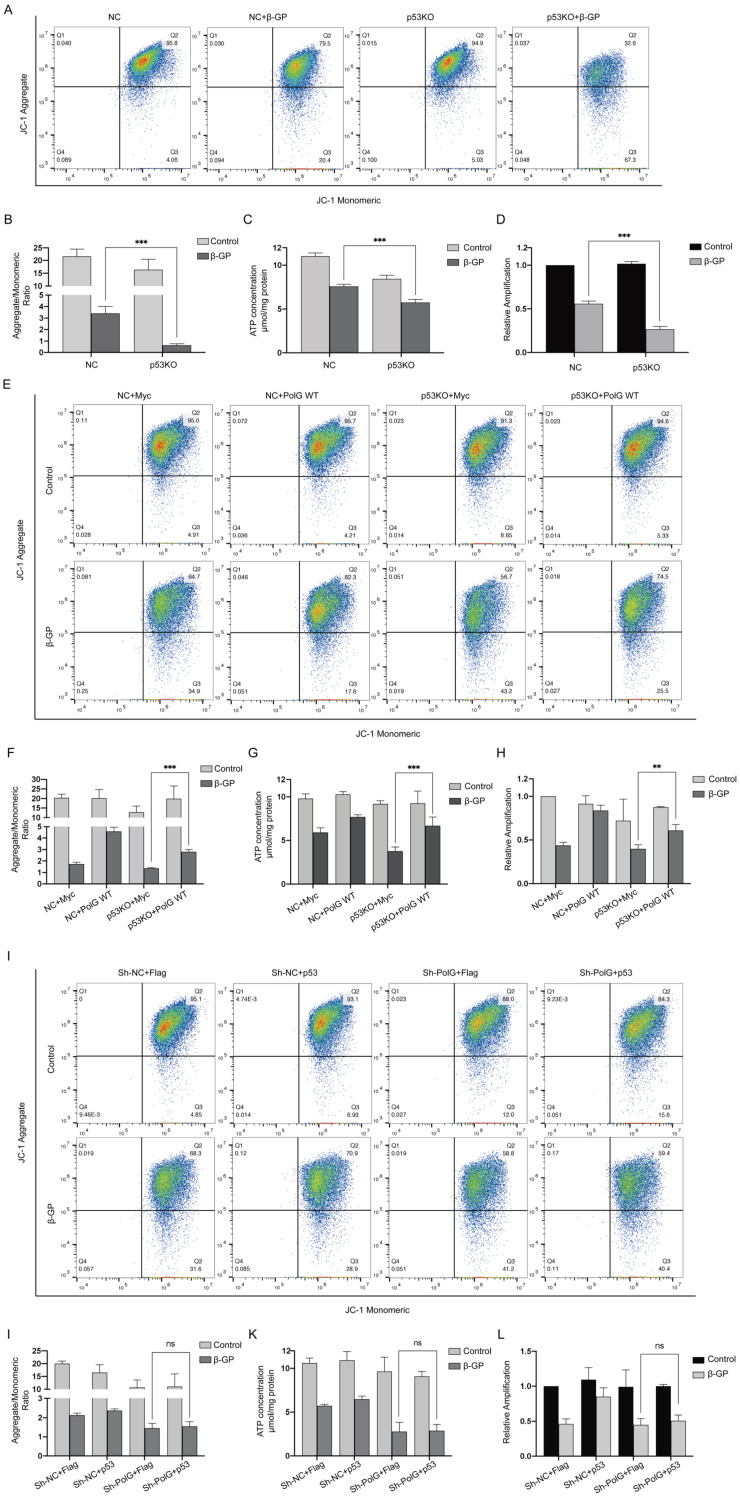
** The stabilization of mitochondrial function by PolG is regulated by p53.** (A-D) NC VSMCs and p53 knockout VSMCs were treated with or without 10mmol β-GP for 5 days, and then detected the mitochondrial function. (A, B) Used flow cytometry to detect MMP level of mitochondria. The potential alteration was calculated as red/green fluorescent intensity ratio. Data were collected from 3 repeat experiment. ***p < .001, ns p>0.05. (C) mtDNA copy numbers of VSMCs. Data were collected from 3 repeat experiment and expressed as the mean ± SD. ***p < .001. (D) ATP contents of VSMCs. Data were collected from 3 repeat experiment and expressed as the mean ± SD. ***p < .001. (E-H) NC VSMCs and p53 knockout VSMCs were transfected with Myc-vector or Myc-tag WT PolG in calcification model, and then detected the mitochondrial function. (E, F) Used flow cytometry to detect MMP level of mitochondria. The potential alteration was calculated as red/green fluorescent intensity ratio. Data were collected from 3 repeat experiment. ***p < .001. (G) mtDNA copy numbers of VSMCs. Data were collected from 3 repeat experiment and expressed as the mean ± SD. ***p < .001. (H) ATP contents of VSMCs. Data were collected from 3 repeat experiment and expressed as the mean ± SD. **p < .005. (I-L) NV VSMCs and PolG knockdown VSMCs were transfected with Flag-vector or Flag-p53 in calcification model, and then detected the mitochondrial function. (I, J) Used flow cytometry to detect MMP level of mitochondria. The potential alteration was calculated as red/green fluorescent intensity ratio. Data were collected from 3 repeat experiment. ns p>0.05. (K) mtDNA copy numbers of VSMCs. Data were collected from 3 repeat experiment and expressed as the mean ± SD, ns p>0.05. (L) ATP contents of VSMCs. Data were collected from 3 repeat experiment and expressed as the mean ± SD. ns p>0.05.

As we know, p53 played an important role in stabilizing and repairing DNA and maintaining mitochondrial function 29, 30, so we constructed p53 knockout VSMCs and treated with β-GP and tried to clarify whether p53 participated the PolG related stabilization of mitochondrial function. We found p53 knockout cell had more severe mitochondrial dysfunction after calcification stimulation (Figure 5A-D). To further clarify the regulation effect between p53 and PolG, we transfected PolG and p53 to p53 knockout and PolG knockdown VSMCs in β-GP induced calcification model respectively. After overexpressed PolG to p53 knockout VSMCs, we found PolG partly recovered injured mitochondrial function contained decreased MMP, reduced mtDNA copy numbers and ATP production (Figure 5E-H). However, we didn't observe obvious improvement of mitochondria dysfunction after transfecting p53 to PolG knockdown VSMCs calcification model (Figure 5I-L).

These results suggested the process of PolG improved mitochondrial dysfunction was regulated by p53.

### p53-PolG D257A complex cannot respond to calcification stimulation to maintain mitochondrial function

**Figure 6:**
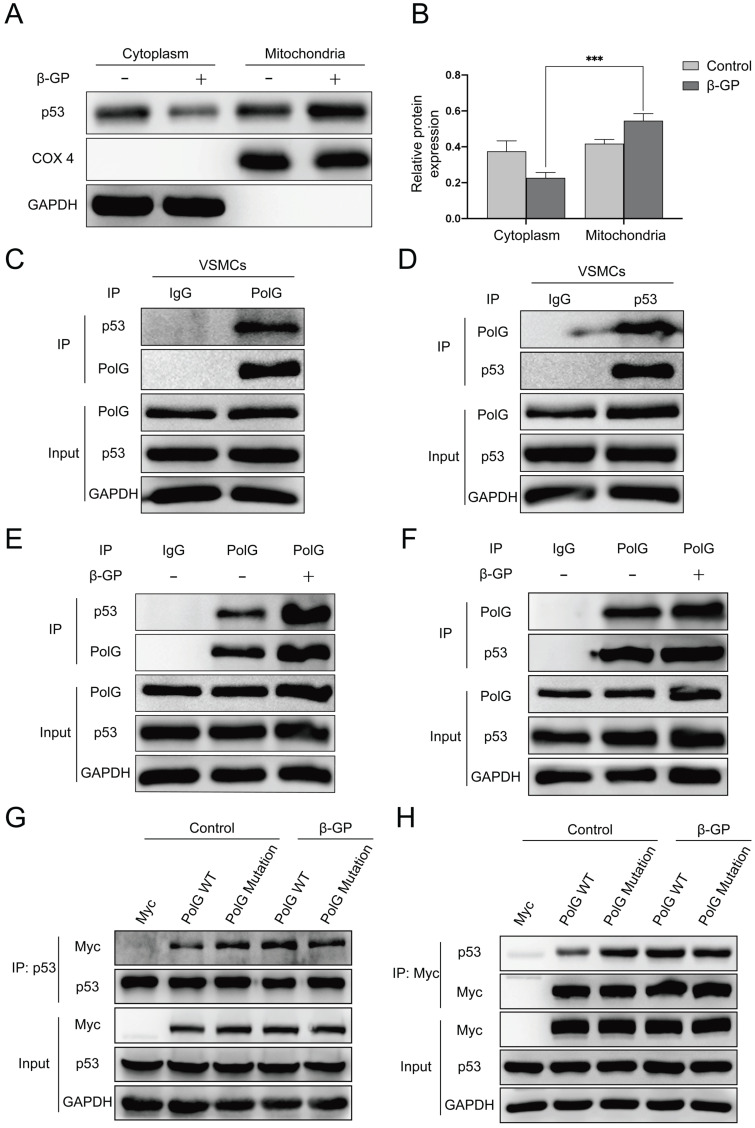
** p53-PolG D257A complex cannot respond to calcification stimulation to maintain mitochondrial function.** (A, B) VSMCs were treated with 10mmol β-GP for 5 days, then harvested the cells and extracted mitochondria. Conducted Western blot assay to detected expression of p53, COX 4 and GAPDH. COX 4 was set as reference of mitochondrial proteins, and GAPDH was set as reference of cytoplasm proteins. Data were collected from 3 repeat experiment and expressed as the mean ± SD. ***p < .001. (C, D) CO-IP assay was conducted by using anti‐PolG or anti‐p53 antibody or negative control IgG and Protein A/G agarose beads to enrichment target proteins. The resulting precipitates were subjected to Western blot analysis with anti‐p53 or anti‐PolG. A portion of the whole-cell lysate was also subjected to WB analysis as input. (E, F) The VSMCs were treated with or without 10mmol β-GP for 5 days, then conducted CO-IP assay to detect interaction alteration of PolG and p53. CO-IP assay was conducted by using anti‐PolG or anti‐p53 antibody or negative control IgG and Protein A/G agarose beads to enrichment target proteins. The resulting precipitates were subjected to Western blot analysis with anti‐p53 or anti‐PolG. A portion of the whole-cell lysate was also subjected to WB analysis as input. (G, H) The VSMCs were transfected with Myc-vector, Myc-tag WT PolG or mutant PolG, treated the cells with or without 10mmol β-GP. Harvested the cells and conducted CO-IP assay to clarify interaction alteration of different types of PolG and p53. CO-IP assay was conducted by using anti‐PolG or anti‐p53 antibody or negative control IgG and Protein A/G agarose beads to enrichment target proteins. The resulting precipitates were subjected to Western blot analysis with anti‐p53 or anti‐PolG. A portion of the whole-cell lysate was also subjected to WB analysis as input.

To further explore the molecular mechanism of calcification regulated by p53 and PolG, we extracted mitochondria from cell and observed p53 translocated to mitochondria from cytoplasm when the mitochondria got injury (Figure 6A-B). Besides, we performed CO-IP assay and revealed p53 could interaction with PolG to execute mtDNA repairs (Figure 6C-D). In addition, we observed the interaction between p53 and PolG was significantly enhanced in β-GP induced calcification (Figure 6E-F). To figure out whether different effect of WT and mutant PolG was caused by interaction with p53, we exogenous overexpressed WT and mutant PolG plasmids in normal condition and calcification condition respectively, and then we conducted CO-IP to evaluate the interaction between p53 and PolG. To our surprise, we observed the interaction between WT PolG and p53 was significantly enhanced under calcification condition, while the interaction between mutant PolG and p53 wasn't enhanced or even attenuated by calcification stimulation (Figure 6G-H).

These alterations and difference suggested p53-PolG D257A complex couldn't respond to calcification stimulation, leading to mutant PolG was fail to maintain mitochondrial function.

### PolG D257A mutation attenuates the stabilizing effect of p53-PolG complex on mitochondria and aggravates vascular calcification

**Figure 7:**
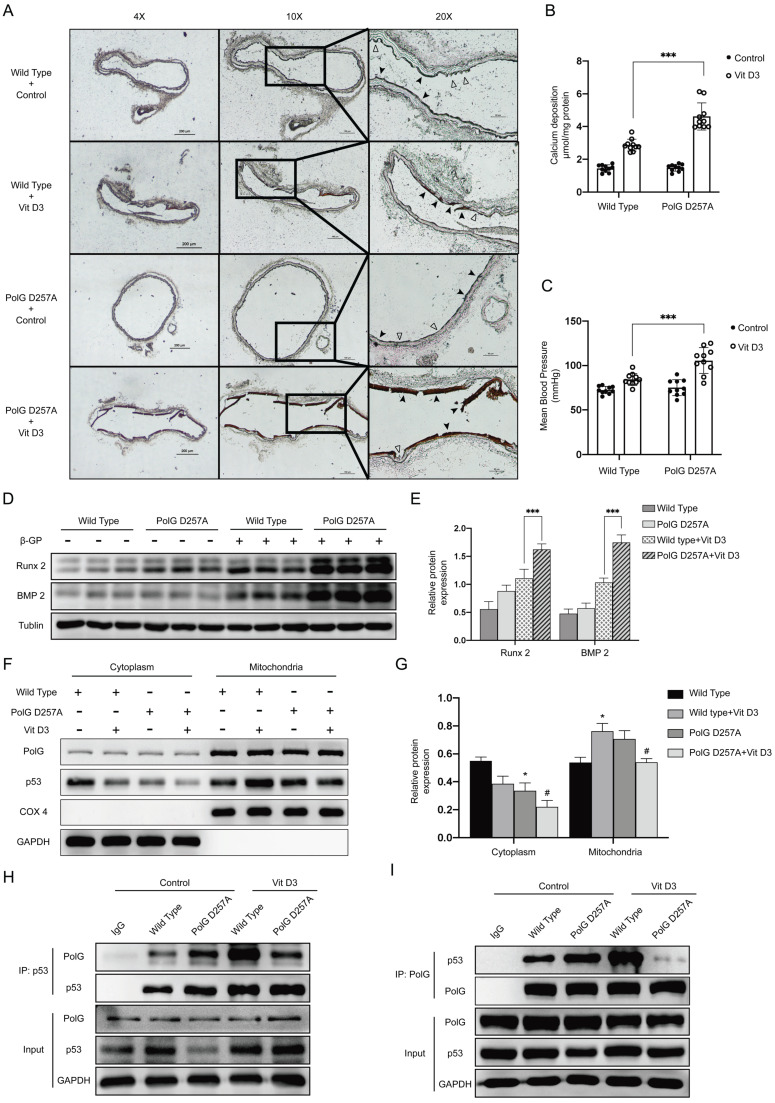
** PolG D257A mutation attenuates the stabilizing effect of p53-PolG complex on mitochondria and aggravates vascular calcification.** The WT mice and POLGD257A/D257A mice was treated with or without 8*10^5 UI/Kg Vit D3 respectively to construct calcification model. (A) Von-Kossa staining of mice aortas. Black arrows pointed to deposition of calcium or calcified VSMCs, and hollow arrows pointed to the normal or pathology structure of vessels. (B) Calcium deposition of aortas, data were collected from 10 individual mice (10 mice in one experiment group) and expressed as the mean ± SD. ***p < .001. (C) MBP of mice in different treatment groups (n=10). Data were expressed as the mean ± SD. ***p < .001. (D, E) Sacrificed mice and isolated the aortas, then conducted WB assay to detected expression of Runx 2, BMP 2 and Tublin, Tublin was set as a loading control. Data were collected from 10 individual mice and expressed as the mean ± SD. ***p < .001. (F, G) Sacrificed mice and isolated the aortas, then extracted mitochondria from them. Conducted WB assay to detected expression of p53, COX 4 and GAPDH. COX 4 was set as reference of mitochondrial proteins, and GAPDH was set as reference of cytoplasm proteins. Data were collected from 10 individual mice and expressed as the mean ± SD. the negative control was set as the panel which were not treated with Vit D3 in each location and mice type, ^#^p < .05. *p < .05, ns p>0.05. (H, I) CO-IP assay to clarify interaction alteration of different types of PolG and p53. CO-IP assay was conducted by using anti‐PolG or anti‐p53 antibody or negative control IgG and Protein A/G agarose beads to enrichment target proteins. The resulting precipitates were subjected to Western blot analysis with anti‐p53 or anti‐PolG.

Finally, we performed calcification *in vivo* to confirm our hypothesis. After calcification protocol in mice, we isolated the aortas and conducted Von-kossa staining and calcium deposition detection respectively to compare calcification status between WT mice and *PolG*^D257A/D257A^ mice. In *PolG*^D257A/D257A^ mice, we observed more severe calcification than WT mice, the elastic layer of aortas took place completely damage and the structure of smooth muscle was disappeared, the vascular intima became smooth, the aortas became stiffness and fragility leading to multiple ruptures (Figure 7A). The quantification result of calcium deposition was consistent with previous staining tendency, a large amount of calcium was deposited in the aortas of *PolG*^D257A/D257A^ mice (Figure 7B). We also measured MBP to evaluate the stiffness degree of mice and found *PolG*^D257A/D257A^ mice had a significantly higher MPB which confirmed the staining results (Figure 7C). Besides, the expression of Runx 2 and BMP2 was consistent with previous results (Figure 7D-E). To confirm our previous molecular mechanism, we extraction mitochondria from aortas of WT mice and *PolG*^D257A/D257A^ mice with or without Vit D3 treated. After detecting expression and location of p53, we found calcification stimulation could promote p53 translocate into mitochondria in WT mice, while we observed the same stimulation couldn't promote mitochondria of *PolG*^D257A/D257A^ mice to recruit more p53 (Figure 7F-G). We also conducted CO-IP experiment to clarify the interaction between p53 and PolG *in vivo*, and we observed calcification stimulation significantly enhanced interaction between p53 and PolG in WT mice (lane 4 vs. lane 2). While in the D257A mice with Vit D3 treatment, we noticed the binding between p53 and PolG was no significant change or even lower than that without calcification stimulation (lane 5 vs. lane 3), which mean the p53-PolG complex wasn't activated or even reduced in PolG D257A/D257A mice under calcification stimulation (Figure 7H-I).

These results suggested p53-PolG complex wasn't activated by calcification stimulation in *PolG*^D257A/D257A^ mice, leading to PolG D257A mutation attenuated the stabilizing effect of mitochondria and aggravated vascular calcification.

## Discussion

**Figure 8:**
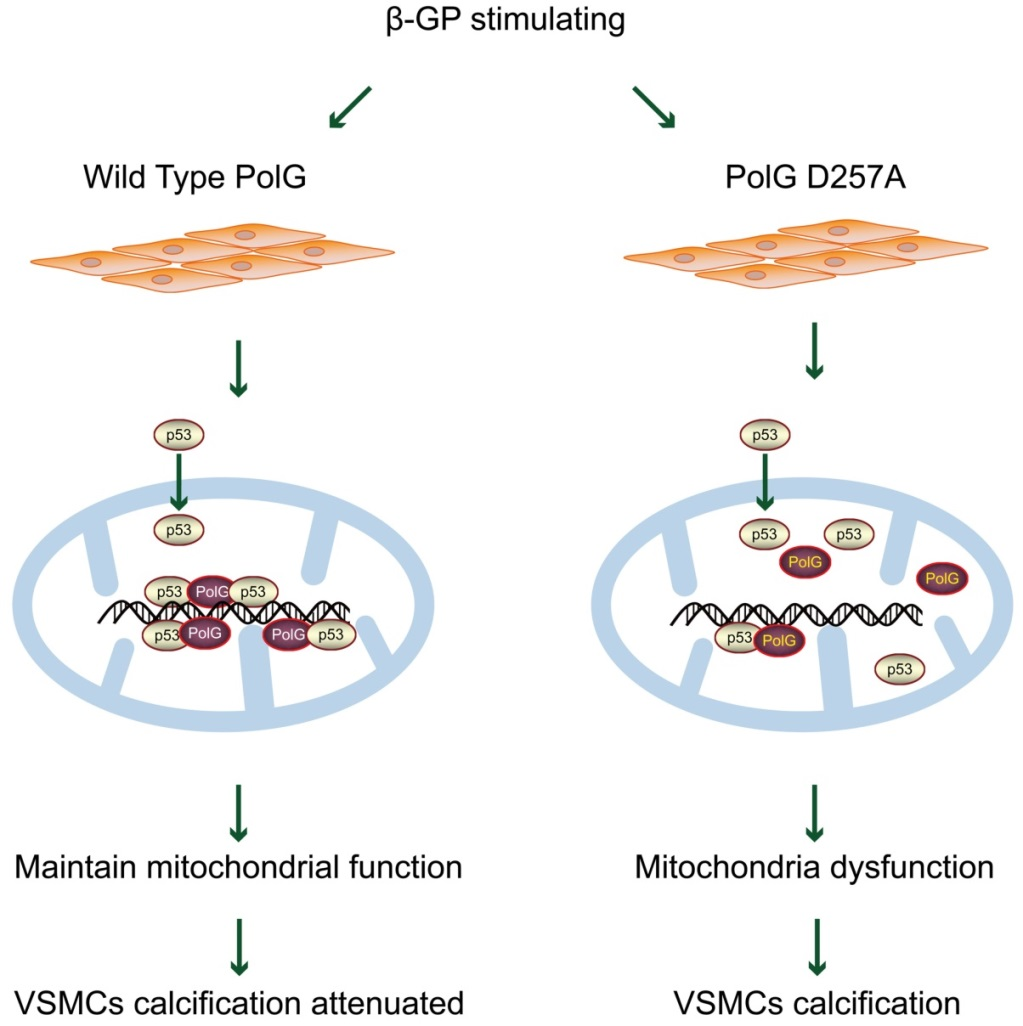
** Model for PolG rescues mitochondrial function and inhibits calcification by interacted with p53.** PolG can recruit and interact with p53 to keep mtDNA stabilization, and these behaviors can be enhanced by calcification stimulation. While mutant PolG fails to form enhanced PolG-p53 complexes in response to calcification, eventually leading to mitochondrial dysfunction and severe calcification. See texts for details.

In present study, we established a potential mechanism that PolG could maintain and rescue mitochondrial function to resist exogenous calcification stimulation. Our finding shown that in response to β-GP induced calcification, PolG recruited p53 into mitochondria and formed p53-PolG complex to repair damaged mitochondria and attenuate calcification degree. Meanwhile, we compared WT and mutant PolG *in vitro* and *in vivo*, and we observed mutant PolG or PolG^D257A/D257A^ mice failed to activate mitochondria repair mechanism, leading to more severe calcification eventually, which further confirmed our conclusions (Figure 8). Thus, we believed PolG was a potential therapeutic target against calcification, providing a novel insight to clinical treatment.

PolG was the most important polymerase of mitochondria and responsible for replication and correction of mtDNA, which determined the quality and quantity of PolG was closely related to mitochondrial function. Some previous research had descripted various serve pathology alterations in PolG^D257A/D257A^ mice 1, 10, 31-33. Some studies observed and compared the differences between WT and heterozygous, WT and homozygous respectively. They found heterozygous mice had some difference at the molecular level compared with WT mice, but there was no difference in phenotype or life span between heterozygous and WT mice. However, homozygous mice all showed significant pathology damage in mitochondria dysfunction and phenotypes 13, 34. For example, D257A mutant of PolG directed reduced the content of electron transport chain (ETC) complexes I, III and IV which could promote skeletal muscle apoptosis and sarcopenia 35, and Tadafumi *et.al* constructed same model in PolG^+/D257A^ mice which were recognized had no difference in phenotypes of WT mice 36, 37, while they found the motor dysfunction caused by heterozygous mutation PolG was significant milder than that in homozygous mutation mice 38. These results suggested that PolG could improve mitochondrial dysfunction induced pathology injury which caused by exogenous stimulation. Vascular calcification was thought as senescence related degenerative damage and involved in various end-stage of chronic diseases which has got more attention in recent years. The causal relationship between calcification and mitochondria damage was still controversial 39, but mitochondrial dysfunction such as declined ATP production or decreased MMP were accompanied with vascular calcification 19, 40, 41. Some studies also found mitochondria related oxidative stress damage could activate phosphatidylinositol 3-kinase/protein kinase-B (PI3K/PKB)signaling pathway 42, 43 and nuclear factor-kappa B (NF-κB) signaling pathway to promote transition of VSMCs into osteogenetic phenotype 44. Thus, we speculated whether PolG could still execute the same effect in the calcification condition. To verify our hypothesis, we respectively overexpressed and knockdown PolG to VSMCs in calcification model, and we observed calcification indeed brought severely damage to mitochondria and exogenous expression of PolG could obviously improve calcification degree induced by β-GP and recovered mitochondrial function *in vitro* which was consistent with previous conclusions and further confirmed our hypothesis that PolG could stabilize and rescue mitochondrial function and further to resist the calcification damage.

Previous studies have observed various pathology phenomenon caused by PolG D257A mutation were significantly rescued by multiple methods of improving mitochondrial function, such as exogenous overexpressed mitochondrial targeted catalase (mCAT) could extend life span of PolG^D257A/D257A^ mice and relieve age-dependent cardiomyopathy by which reducing mitochondrial oxidative stress damage or ROS induced mitochondrial damage 45, 46. Thus, we supposed to guarantee mitochondrial function by maintain quality and quantity of mtDNA. p53 is an important DNA damage repair factor which involved in the stabilization of genome DNA 47. PolG is recognized as the only on polymerase of mtDNA, so we supposed whether p53 was involved in the PolG related mtDNA repair process. So we transfected PolG and p53 into p53 knockout cells and PolG knockdown cells respectively, and detected their mitochondrial function. When mtDNA is damaged under calcification stimulating, as we known, PolG is the molecule that really plays the role of recognition, correction, replication and polymerization of mtDNA. And p53 is the key molecule which could stop the replication of abnormal mtDNA timely and enhance the efficiency of PolG to identify and correct abnormal mtDNA, reducing the accumulation of damaged or abnormal mtDNA. So in the whole process, p53 didn't directly participate in the replication and correction process of mtDNA, and PolG is the direct executive molecule of mtDNA polymerization and damage repairing. p53 only enhances this process and can indirectly regulate this process, so it can be considered as an upstream molecule of PolG. Therefore, after transfecting PolG into p53 knockout cells, even if the efficiency of PolG in correcting the abnormal mtDNA was reduced due to the lack of p53, but the excessive expression of PolG partially could made up for this defect, so the mitochondrial function was recovered. However, there was no significant increase in the level of PolG after transfecting p53 into PolG knockdown celle, so it didn't significantly restore mitochondrial function. Nextly, we compared the location of p53 and confirmed p53 was activated and recruited into mitochondria, and further interacted with PolG to repair and stabilize mtDNA. Meanwhile, we also found the translocation of p53 and the interaction with PolG could be both enhanced by calcification stimulation *in vitro* and *in vivo*. So, we believed p53-PolG complexes could be activated to repair mtDNA and maintain mitochondrial function in response to calcification. Some mechanism studies also shown PolG played a crucial role in stabilization of mitochondrial function by p53-dependent pathway or interacted with p53 17, 48, which further confirmed our conclusions that p53-PolG interaction played an indispensable role in stabilization of mitochondrial function and protected cells from exogenous damage stimulation. A previous study shown 3'-5' exonuclease activity of PolG was necessary to maintain the integrity of mtDNA 49. Based on this conclusion, we compared WT mice and PolG^D257A/D257A^ mice to identify whether Vit D3 induced different outcomes were caused by mutant PolG related repair capability deficiency. To our surprise, compared with WT PolG, the p53-PolG complex didn't respond to calcification stimulation in PolG^D257A/D257A^ mice, leading to the interaction complex was fail to activate the effect of repairing and rescuing mitochondrial damage. Thus, we believed PolG could enhance the interaction with p53 in calcification condition to improve mtDNA damage, maintain the stability of mitochondrial function and resist calcification damage. The role of PolG as a multi-functional enzyme depends on three conserved domains, one of which is the domain of exonuclease performing proofreading function. The mutation of D257A happened to occur in this domain, resulting in the deficiency of proofreading ability. Compared with genomic DNA, p53 forcibly stop the replication when DNA was damaged or mutations were detection, so as to further recruit corresponding exonucleases for DNA repairing. Therefore, our results ensure us to hypothesize that this 257th amino acid residue site could affect the recognition and interaction ability of PolG with p53. Damaged mtDNA could initiate repairing effect of PolG, at this time, the participation of p53 could greatly improve and guarantee the proofreading efficiency of PolG on mtDNA. When the damaged degree of mtDNA beyond the correction ability limits of PolG, due to p53 couldn't be bind with mutant PolG, and as the consequence, p53 was unable to amplify the correction function of PolG to damaged mtDNA which resulting in the accumulation of damaged or mutation mtDNA under the condition of exogenous damage stimulation, and eventually presented serious calcification. Thus, the integrity function of PolG was also an important prerequisite for recovering mitochondrial function and resisting the calcification damage.

We also had some limitations in spreading our conclusions. First, we did not collect calcified vessels samples of human beings to verify our conclusion. On the one hand, it was difficult for us to obtain calcified aortas, Meanwhile, *PolG* had a variety of mutation sites, but we only focused on the proof-reading-deficient of PolG, so we directly chose the corresponding animal model to ensure the universality and accuracy of our conclusions *in vivo*. In addition, we didn't construct p53^-/-^PolG^D257A/D257A^ mice to further improve our molecular mechanism research. Considering the premature aging and reproductive impairment characteristics of PolG^D257A/D257A^ mice, it was difficult to construct p53^-/-^PolG^D257A/D257A^ mice. Therefore, we have fully proved our conclusion *in vitro* and believed our conclusions were credible.

In summary, our study suggested for the first time that PolG could attenuate β-GP induced calcification by recruiting and interacting with p53 to keep stabilization of mtDNA and maintain mitochondrial function. Meanwhile, we revealed this effect was determined by integrity function of PolG, especially the 3'-5' exonuclease activity to guarantee the enhanced interaction of p53 and PolG in response to calcification stimulation. Thus, we believed PolG was a potential therapeutic target against calcification, providing a novel insight to clinical treatment.

## Abbreviations

β-GP: beta-glycerophosphate
BMP 2: bone morphogenetic protein 2
CO-IP: co-immunoprecipitation
DMEM: Dulbecco's modified Eagle's medium
FBS: fetal bovine serum
M: mean
MBP: mean blood presure
mCAT: mitochondrial targeted catalase
MMP: mitochondrial membrane potential
mtDNA: mitochondrial DNA
NC: negative control
NF-κB: nuclear factor-kappa B
PBS: phosphate buffered saline
PCR: polymerase chain reaction
PI3K: phosphatidylinositol 3-kinase
PKB: protein kinase-B
PolG: DNA polymerase gamma
Runx 2: runt-related transcription factor 2
SD: standard deviation
VC: vascular calcification
Vit D3: vitamin D3
VSMCs: Vascular smooth cells
WB: Western Blot
WT: Wild Type
